# Breast Cancer Care in South India: Is Practice Concordant With National Guidelines?

**DOI:** 10.1200/JGO.19.00052

**Published:** 2019-07-01

**Authors:** D.K. Vijaykumar, Sujana Arun, Aswin G. Abraham, Wilma Hopman, Andrew G. Robinson, Christopher M. Booth

**Affiliations:** ^1^ Amrita Institute of Medical Sciences and Research Centre, Cochin, India; ^2^ Kingston General Hospital Research Institute, Kingston, Ontario, Canada; ^3^ Queen’s University Cancer Research Institute, Kingston, Ontario, Canada; ^4^ Queen’s University, Kingston, Ontario, Canada

## Abstract

**PURPOSE:**

The National Cancer Grid (NCG) of India has recently published clinical practice guidelines that are relevant in the Indian context. We evaluated the extent to which breast cancer care at a teaching hospital in South India was concordant with NCG guidelines.

**METHODS:**

All patients who had surgery for breast cancer at a single center from January 2014 to December 2015 were included. Demographic, pathologic, and treatment characteristics were extracted from the electronic medical record. Patients were classified as being concordant with six elements selected from the NCG guideline. The indicators related to appropriate use of sentinel lymph node (SLN) biopsy, lymph node harvest, adjuvant radiotherapy, adjuvant chemotherapy, human epidermal growth factor receptor 2 (HER2) testing, and delivery of adjuvant trastuzumab.

**RESULTS:**

A total of 401 women underwent surgery for breast cancer; mean age (standard deviation) was 57 (12) years. Lymph node involvement was present in 47% (188 of 401) of the cohort; 23% (94 of 401) had T1 disease. Ninety-two percent (368 of 401) underwent radical modified mastectomy. SLN biopsy was performed in 75% (167 of 222) of eligible patients. Eighty percent (208 of 261) of patients with a positive SLN biopsy or no SLN biopsy had a lymph node harvest of more than 10. Adjuvant chemotherapy with an anthracycline and a taxane was delivered to 67% of patients (118 of 177) with node-positive disease. Adjuvant radiotherapy was delivered to 84% (180 of 213) of patients with breast-conserving surgery, T4 tumors, or 3+ positive lymph nodes. Fluorescent in situ hybridization testing was performed in 59% of patients (43 of 73) with 2+ HER2-positive lymph nodes on immunohistochemistry. Among patients with HER2 overexpression, 40% (36 of 91) received adjuvant trastuzumab.

**CONCLUSION:**

Concordance with NCG guidelines for breast cancer care ranged from 40% to 84%. Guideline concordance was lowest for those elements of care associated with the highest direct costs to patients.

## INTRODUCTION

Clinical practice guidelines (CPGs) in oncology serve several important roles. They promote high-quality evidence-based care delivery, help standardize treatment and reduce unnecessary variations in care, and serve to identify gaps in knowledge and areas where future evidence generation is a priority. In addition to directing clinical care, CPGs also offer a mechanism to measure quality of care in which practice observed in the real world can be compared with guideline recommendations.

Since the early 2000s, it has become increasingly recognized that health system priorities and CPGs should be context specific, taking into account the available resources for cancer control. At a broad level, this acknowledges that cancer control priorities and recommended treatments will, by necessity, be different between a high-income country (HIC) and a low-income country. The Breast Health Global Initiative (BHGI) has developed a methodology for the creation of resource-stratified practice guidelines.^1,2^ Working in the field of breast cancer, the BHGI has published a series of guidelines spanning early detection, diagnosis and pathology, treatment, and health care systems that are specifically tailored across four resource tiers: basic, limited, enhanced, and maximal. Subsequent to this, the BHGI created guideline implementation quality metrics that were linked to the resource stratification system.^3^ Since then, a resource-stratified guideline has been developed by the American Society of Clinical Oncology (ASCO) for cervical cancer.^4^ Other groups, including the World Bank and the National Comprehensive Cancer Network, have also used resource-stratified frameworks to develop practice guidelines.^2^

**CONTEXT**

**Key Objective**
The National Cancer Grid (NCG) of India recently published clinical practice guidelines that are relevant in the Indian context. We evaluated the extent to which breast cancer care at a teaching hospital in South India was concordant with six specific elements of the NCG guideline.
**Knowledge Generated**
Sentinel lymph node biopsy was performed on 75% of eligible patients. Eighty percent of patients with nodal dissection had a lymph node harvest of more than 10 nodes. Adjuvant chemotherapy with an anthracycline and a taxane was delivered to 67% of patients with node-positive disease. Adjuvant radiotherapy was delivered to 84% (180 of 213) of eligible patients. Fluorescent in situ hybridization testing for human epidermal growth factor receptor 2 status was performed in 59% of patients. Among patients with human epidermal growth factor receptor 2 overexpression, 40% (36 of 91) received adjuvant trastuzumab.
**Relevance**
Concordance with NCG guidelines for breast cancer care ranged from 40% to 84%. Guideline concordance was lowest for those elements of care associated with the highest direct costs to patients.

In 2017, the National Cancer Grid (NCG) of India released a series of guidelines for management of more than 70 common cancers in the Indian context.^5^ The NCG aims to facilitate efforts in cancer control, research, and education. A particular emphasis is the development of uniform standards of cancer care across India.^6-8^ Over the past 6 years, it has grown to a large network of 143 cancer centers, research institutes, patient advocacy groups, charitable organizations, and professional societies. Incorporating virtually all stakeholders of cancer care in India, the NCG has become a strong, unified, and powerful voice within the Indian health system. The NCG has developed evidence-based and context-appropriate guidelines for the management of cancer in India.^5^ This massive undertaking engaged experts from multiple disciplines across India and included patient representatives. The guidelines are evidence based, practical, and succinct; management of most cancers are summarized in two to five pages. It is envisioned that NCG members will eventually contribute center-level data that will facilitate measurement of care against the NCG guidelines, which will also continue to be updated. Development of the necessary data-sharing agreements and health information technology systems are under way. However, an earlier understanding of the extent to which NCG guidelines are followed in routine practice will require individual center analyses. To date, there are limited reports from low- and middle-income countries (LMICs) that describe treatment concordance with CPGs for any cancer. To address this gap in knowledge and to gain insight into the applicability of the NCG guidelines to Indian cancer centers, we undertook a study to describe practice and concordance across six key recommendations from the NCG guidelines for breast cancer at a single high-volume center in South India.

## METHODS

### Study Setting

This was a retrospective cohort study of all women who underwent surgery for breast cancer from 2014 to 2015 at Amrita Institute of Medical Sciences (AIMS). AIMS is a private, 1,200-bed tertiary care teaching hospital located in the southern Indian city of Kochi. AIMS is recognized as a leading hospital in India and offers courses at the undergraduate and postgraduate levels. The hospital has 25 operating theaters and performs approximately 20,000 surgeries per year. The Department of Breast and Gynecologic Oncology provides comprehensive outpatient, inpatient, and surgical care to all women with breast and gynecologic cancers. The Department includes two consultants in surgical oncology, four consultants in medical oncology, five consultants in radiation oncology postgraduate trainees in all oncology disciplines.

All patients who underwent surgery for breast cancer between January 1, 2014, and December 31, 2015, were identified from a review of the Operating Theater register. Patients with benign breast diseases, other nonmalignant pathologies (ie, granulomatous mastitis), and nonbreast cancer malignancies (ie, sarcoma) were excluded. The study was approved by the AIMS institutional review board.

### Data Sources

Demographic, pathologic, and treatment characteristics were extracted from the electronic medical record by a trained research assistant. Quality assurance and extensive data review were performed by the principal investigator (D.K.V.). To explore concordance of clinical practice with guideline recommendations, we classified each patient as being concordant or not concordant with six elements selected from the NCG indicators. The six indicators were selected on the basis of relevance to routine clinical practice, common elements of care, and feasibility of measurement from the electronic medical record. Two indicators related to surgical care and one each to adjuvant chemotherapy, adjuvant radiotherapy (RT), advanced diagnostic testing, and targeted therapy. The six indicators were:

1. Patients with clinical and radiologic stage N0 should receive sentinel node biopsy.
2. Patients having an axillary dissection should have at least 10 dissected nodes.
3. Patients with three or more positive nodes or with breast conservation or stage T4 tumors should receive postoperative adjuvant RT.
4. Patients with node-positive disease should receive adjuvant chemotherapy with an anthracycline and a taxane.
5. Patients with humman epidermal growth factor receptor 2 (HER2) 2+ on immunohistochemistry (IHC) should have fluorescent in situ hybridization (FISH) testing.
6. Patients with HER2 3+ or FISH showing amplification should receive trastuzumab.

Although the NCG guidelines do not list a specific adjuvant chemotherapy regimen, the Indian Council of Medical Research uses an anthracycline plus a taxane; this combination was therefore considered to be compliant with guidelines.^9^ The primary investigator (D.K.V.) and a senior postgraduate trainee (A.G.A.) reviewed the indicators for each patient to determine treatment concordance.

### Statistical Analysis

Data were entered into an Excel spreadsheet (Microsoft Corporation, Redmond, WA) and imported into IBM SPSS (version 24.0 for Windows; Armonk, NY) for statistical analysis. Data were primarily analyzed descriptively, including means and standard deviations for continuous data, such as age, and frequencies and percentages for categorical data. Concordance with guidelines was also analyzed descriptively using subsets as required.

## RESULTS

### Study Population

From 2014 to 2015, 401 women underwent surgical resection of breast cancer. The mean age was 57 years (standard deviation, 12 years; range 23 to 92 years); 8% of women (33 of 401) were younger than 40 years of age (Table 1). The decade of peak incidence was 50 to 59 years. Fifty-five percent of patients (222 of 401) had T2 primary tumors; only 23% (94 of 401) had T1 disease. Lymph node involvement was present in 47% (188 of 401) of the study population. Estrogen receptors (ERs) and progesterone receptors (PRs) were expressed in 69% (275 of 398) and 55% (222 or 397) of patients, respectively. HER2 overexpression was identified (using IHC) in 23% of patients (91 of 401). Fourteen percent of patients (54 of 383) with ER/PR/HER2 testing were found to have triple-negative disease. The distribution of biologic subtype was 26% luminal A (104 of 401), 32% luminal B (127 of 401), 22% HER2 (90 of 401), 17% basal (67 of 401), and 3% unknown (13 of 401).

**TABLE 1:**
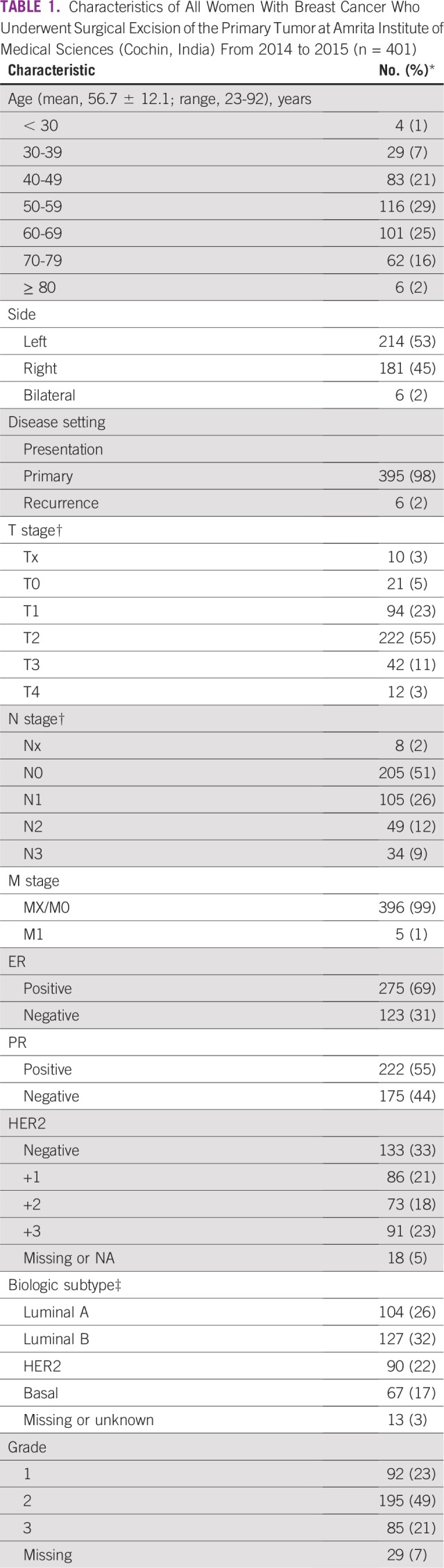
Characteristics of All Women With Breast Cancer Who Underwent Surgical Excision of the Primary Tumor at Amrita Institute of Medical Sciences (Cochin, India) From 2014 to 2015 (n = 401)

### Treatment Delivery

The vast majority of patients (92%; 368 of 401) underwent modified radical mastectomy (Table 2). Neoadjuvant and adjuvant chemotherapy were delivered to 16% (63 of 401) and 59% (238 of 401) of patients, respectively; 44% of patients (178 of 401) received adjuvant RT. Adjuvant hormonal therapies with tamoxifen and letrozole were given to 27% (109 of 401) and 35% (142 of 401) of patients, respectively.

**TABLE 2:**
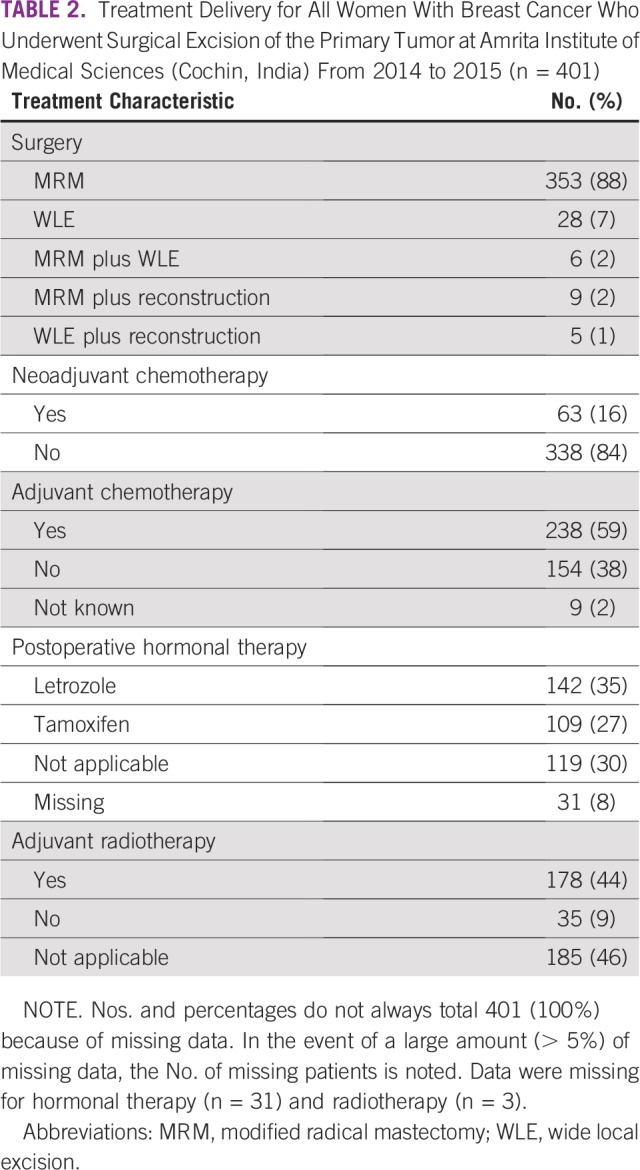
Treatment Delivery for All Women With Breast Cancer Who Underwent Surgical Excision of the Primary Tumor at Amrita Institute of Medical Sciences (Cochin, India) From 2014 to 2015 (n = 401)

### Concordance With NCG Guidelines

As listed in Table 3, concordance with NCG guidelines ranged from 40% (adjuvant trastuzumab) to 84% (adjuvant RT). Sentinel lymph node (SLN) biopsy was performed in 75% of patients (167 of 222) in whom it was indicated. Eighty percent of patients (208 of 261) with a positive SLN biopsy or no SLN biopsy had greater than 10 lymph nodes sampled at the time of surgery. Adjuvant chemotherapy with an anthracycline and a taxane was delivered to 67% of patients (118 of 177) with lymph node–positive disease. Adjuvant RT was delivered to 84% of patients (180 of 213) with breast-conserving surgery (BCS), T4 tumors, of 3+ positive lymph nodes. FISH testing was performed in 59% of patients (43 of 73) with 2+ HER2 on IHC. Finally, among patients with 3+ HER2 overexpression on IHC or FISH-positive results, 40% (36 of 91) received adjuvant trastuzumab.

**TABLE 3:**
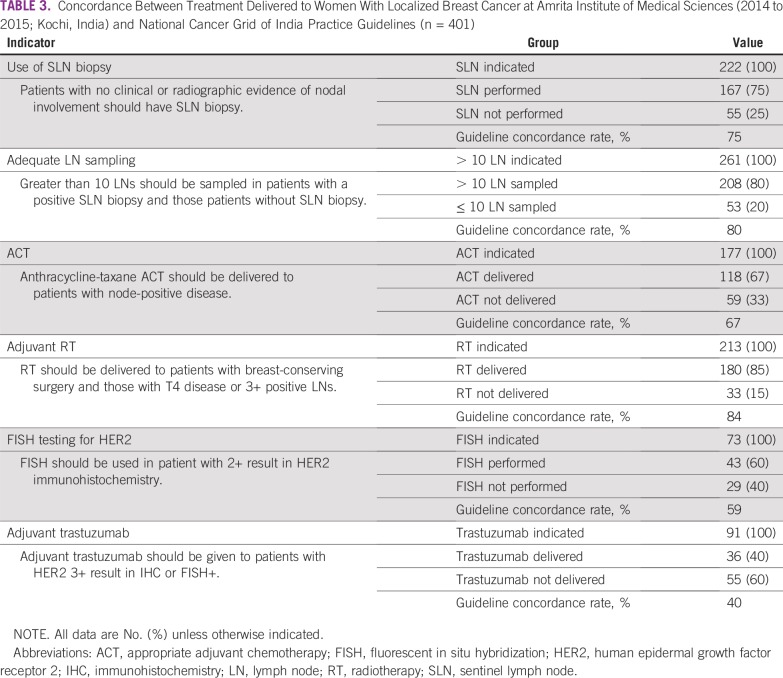
Concordance Between Treatment Delivered to Women With Localized Breast Cancer at Amrita Institute of Medical Sciences (2014 to 2015; Kochi, India) and National Cancer Grid of India Practice Guidelines (n = 401)

## DISCUSSION

In this study, we explored the extent to which clinical practice for surgically resected breast cancer was consistent with evidence at one of India’s leading institutions. Several important findings emerged. First, compared with cohorts from HICs, patients in this study had more advanced tumors and were more likely to have lymph node involvement. Second, ER/PR status and HER2 status seen in this cohort was comparable to reports from HICs. Third, rates of BCS were far lower in this population compared with current rates in HICs. Fourth, concordance with guidelines varied and ranged from 40% to 84%. Finally, the elements of care that were the most discordant with guidelines were tests and therapies that were associated with considerable cost. There are a multitude of factors within the Indian cancer system that likely contribute to practice being discordant with clinical guidelines, including the cost of care (and limited health insurance plans), the lack of drug access programs for patients without the means to pay for therapy, limited oncology workforce capacity in many parts of India, and low health literacy among many segments of the Indian population. It is hoped that recent initiatives, such as the Ayushman Bharat National Health Protection Mission, will reduce the gap between evidence and clinical practice.

Patients in India and other LMICs are more likely to present with advanced disease compared with patients in HICs.^10,11^ In India and other LMICs, it is known that lower socioeconomic status is associated with more advanced breast cancer at the time of diagnosis.^12-14^ The proportion of patients with T1 tumors in our study cohort (23%) was much lower than reports from the United States (61%) and was also lower than many other parts of Asia.^15,16^ The reasons for this are complex and multifactorial, and may include lower health awareness, lack of access to primary care and cancer work-up services, sociocultural barriers, and financial costs. In India, out-of-pocket payments account for approximately 75% of cancer costs and are increasingly a common cause of catastrophic financial expenditures for the patient and family.^17^ These financial barriers further contribute to the problems with delayed diagnosis and incomplete treatment. A comparable proportion of early-stage breast cancer was reported in Latin America (approximately 20%), which shares many of the same challenges as India regarding timely cancer diagnosis.^18^

The rate of BCS in our study population (8%) was far lower than reports from HICs (approximately 60%)^19^ but comparable to other LMICs. Huang et al^20^ evaluated surgical management of more than 18,000 women in China from 1999 to 2013 and reported a BCS rate of 15%. However, in another recent single-center report from South India among 401 surgical patients with breast cancer, Ali et al^21^ reported a BCS rate of 41%. Reasons for the discordant results between the article by Ali et al^21^ and the our study are not known.

In a quality improvement study from Canada, Enright et al^22^ described performance metrics among 28,427 women with early-stage breast cancer, 41% of whom were treated with adjuvant chemotherapy. Seventy-eight percent of women with ER/PR expression were treated with adjuvant hormonal therapy. In another study from Canada, Ashworth et al^23^ found a postlumpectomy RT rate among 74,220 women of 69%. We are not aware of any studies of breast cancer guideline compliance from LMICs.

Within our own cohort, we reviewed clinical records to understand common reasons for noncompliance with practice guidelines. For sentinel node sampling, most of the noncompliance related to technical issues (ie, scar tissue on breast limited uptake in axilla). In some cases, the procedure was not performed because of nonavailability of radio-isotope or technical problems with the gamma probe. Potential reasons for inadequate lymph node harvest related to surgical decision making in some patients and also to the use of neoadjuvant chemotherapy. Some patients with node-positive disease who had not received anthracyline and taxane adjuvant chemotherapy declined treatment because of financial costs, pursuit of alternative therapy, and/or comorbidity, making the risks of adverse effects substantial. It is also notable that an additional 35 patients received other chemotherapy regimens that did not include anthracycline and taxane; therefore, compliance with this indicator was likely higher than reported (ie, may be as high as approximately 87%). The low rates of FISH testing for HER2 status and use of adjuvant trastuzumab was almost uniformly because of financial considerations; in fact, if a patient cannot afford trastuzumab treatment, testing for HER2 status may not be appropriate.

Our study has important limitations that merit comment. This was a single-center study whose results may not be generalizable to other settings. In particular, these data come from a private teaching hospital. It is therefore likely that guideline concordance may be far lower at other hospitals in India, where there are even fewer resources. We also had a priori selected six specific elements of the NCG breast cancer guideline. Concordance with other elements of care was not evaluated in this study. It is notable that the chosen elements of care are consistent with recommendations from the Indian Council of Medical Research and the BHGI/ASCO resource-stratified guidelines.^9,24^ Our study was limited by the fact that the reason for noncompliance was generally not evident from the clinical chart. Finally, it is worth noting that although the data reported in this study describe practice from 2014 to 2015, the NCG guidelines were not published until 2017. However, the specific treatment recommendations included in this study were already firmly established in the literature by 2014. It will be important to understand whether performance at this institution and others throughout India have improved since 2017. An initiative within the NCG is under way to expand this study to include prospective contemporary cohorts at multiple institutions across India. Data from the future study will inform subsequent knowledge translation efforts to close the gaps between evidence and practice.

This study illustrates that women in South India are commonly diagnosed with advanced breast cancer. Rates of BCS are low. Concordance rates with six specific elements of care range from 40% to 84%. Treatment elements with low concordance are associated with substantial financial cost to the patient and family. Future work is needed to better understand the reasons for noncompliance, whether rates are improving, and the extent to which new government funding programs will close the observed gap between evidence and practice.
